# Molecular Characterization of *Staphylococcus aureus* from Patients with Surgical Site Infections at Mulago Hospital in Kampala, Uganda

**DOI:** 10.1371/journal.pone.0066153

**Published:** 2013-06-20

**Authors:** Jeremiah Seni, Freddie Bwanga, Christine F. Najjuka, Patson Makobore, Moses Okee, Stephen E. Mshana, Benson R. Kidenya, Moses L. Joloba, David P. Kateete

**Affiliations:** 1 Department of Medical Microbiology, Makerere University College of Health Sciences, Kampala, Uganda; 2 Department of Surgery, Makerere University College of Health Sciences, Kampala, Uganda; 3 Department of Microbiology and Immunology, Catholic University of Health and Allied Sciences Bugando, Mwanza, Tanzania; 4 Department of Biochemistry and Molecular Biology, Catholic University of Health and Allied Sciences Bugando, Mwanza, Tanzania; University of Iowa, United States of America

## Abstract

**Background:**

The prevalence of Methicillin resistant *Staphylococcus aureus* (MRSA) is progressively increasing globally with significant regional variation. Understanding the *Staphylococcus aureus* lineages is crucial in controlling nosocomial infections. Recent studies on *S. aureus* in Uganda have revealed an escalating burden of MRSA. However, the *S. aureus* genotypes circulating among patients are not known. Here, we report *S. aureus* lineages circulating in patients with surgical site infections (SSI) at Mulago National hospital, Kampala, Uganda.

**Methods:**

A cross-sectional study involving 314 patients with SSI at Mulago National Hospital was conducted from September 2011 to April 2012. Pus swabs from the patients’ SSI were processed using standard microbiological procedures. Methicillin sensitive *Staphylococcus aureus* (MSSA) and MRSA were identified using phenotypic tests and confirmed by PCR-detection of the *nuc* and *mecA* genes, respectively. SCC*mec* genotypes were determined among MRSA isolates using multiplex PCR. Furthermore, to determine lineages, *spa* sequence based-genotyping was performed on all *S. aureus* isolates.

**Results:**

Of the 314 patients with SSI, *S. aureus* accounted for 20.4% (64/314), of which 37.5% (24/64) were MRSA. The predominant SCC*mec* types were type V (33.3%, 8/24) and type I (16.7%, 4/24). The predominant *spa* lineages were t645 (17.2%, 11/64) and t4353 (15.6%, 10/64), and these were found to be clonally circulating in all the surgical wards. On the other hand, lineages t064, t355, and t4609 were confined to the obstetrics and gynecology wards. A new *spa* type (t10277) was identified from MSSA isolate. On multivariate logistic regression analysis, cancer and inducible clindamycin resistance remained as independent predictors of MRSA-SSI.

**Conclusion:**

SCC*mec* types I and V are the most prevalent MRSA *mecA* types from the patients’ SSI. The predominant *spa* lineages (t645 and t4353) are clonally circulating in all the surgical wards, calling for strengthening of infection control practices at Mulago National Hospital.

## Introduction

*Staphylococcus aureus* is a leading causative agent of surgical site infections (SSI) worldwide [1]. *S. aureus* carriage is the most important factor predisposing colonized individuals to subsequent SSI; other factors are longer duration of hospital stay, HIV infection, cancer, and opsonic defects [2], [3]. Surveillance data have shown that in hospital settings methicillin sensitive *Staphylococcus aureus* (MSSA) tends to evolve into methicillin resistant *Staphylococcus aureus* (MRSA) [4], [5]. Globally the prevalence of MRSA is progressively increasing with significant regional variation [4], [5]. Data in most African countries are scarce; in few surveys the prevalence of MRSA has been found to range from as low as 2% to as high as 41% [6], [7], [8], [9], [10]. In Uganda, previous studies at Mulago National Hospital showed that 28.7% of the SSI are due to *S. aureus,* and of these 31.5% are due to MRSA [11].

The association of MRSA with therapeutic challenges, complications, deaths and cost related to longer hospital stay compared with MSSA has been widely documented [12], [13], [14]. More importantly, the multidrug resistant hospital-acquired MRSA (HA-MRSA) strains and their intrinsic resistance to beta-lactam antibiotics confer limited treatment options to the most available and less costly antibiotics in developing countries [11], [15].

Molecular approaches are of paramount importance in showing clonality and spreading patterns of *S. aureus* strains in hospital settings; however, variation exists about their effectiveness, cost and general applicability [16], [17]. PCR based approaches for characterizing Staphylococcal chromosomal cassettes *mec* (SCC*mec*) types and determining sequence polymorphism in the variable X region of the Staphylococcal protein a (*spa*) have shown to be relatively less expensive, easier, less time consuming and have clear discriminatory power in comparison with other approaches, such as multi locus sequence typing (MLST) and pulse field gel electrophoresis (PFGE) [18], [19], [20].

SCC*mec* types can correlate with the classic HA-MRSA (SCC*mec* types I–III) and community associated MRSA (CA-MRSA) (SCC*mec* types IV and V) [21], [22], [23]. Furthermore, the *pvl* gene, which is a virulence factor for severe staphylococcal infections, has also been associated with CA-MRSA [24]. *Spa* sequence typing can show clonality among *S. aureus* and has portability of data with absolute reproducibility which allows internet-based type assignment and worldwide comparison of isolates [25], [26].

Data showing *S. aureus* genotypes circulating among patients with SSI at Mulago National Hospital to guide infection control surveillance remain scanty. Furthermore, whether there is clonal spread of the predominant genotypes is also not known. Using *spa* sequence typing and SCC*mec* genotyping, we present *S. aureus* lineages in patients with SSI at Mulago National Hospital in Kampala, Uganda.

## Materials and Methods

### Study Design and Sampling Procedures

This cross-sectional study was conducted from September 2011 to April 2012 at Mulago National Hospital, the largest hospital located in Kampala, Uganda, with an estimated 1,500 beds. Using the Kish and Lisle formula with prevalence of 28.7%, a sample size of 314 patients with clinical SSI from obstetrics & gynecology, general surgery and orthopedic wards were enrolled [11]. Consenting patients with SSI occurring within 30 days after the operative procedure or within one year if orthopedic implant is in situ were included in the study, while surgical patients with community acquired pyogenic infections such as abscess, furuncle and carbuncle; patients with infection of an episiotomy; and patients with open fractures were excluded.

### Data Collection and Laboratory Procedure

Demographic and clinical characteristics from consenting patients were collected using structured pretested questionnaire and patients’ files. These included age, sex, ward of admission, operation type, extent of SSI, presence of cancer, HIV serostatus, and length of hospital stay.

The infected site was cleaned using normal saline and sterile gauze, then pus was collected by a sterile cotton swab in moist Amies transport media [27]. Two pus samples were collected from each patient. A nasal swab was also taken from each patient for screening of *S. aureus* carriage. The samples were transported within two hours of collection to the clinical laboratories of the Department of Medical Microbiology, Makerere University College of Health Sciences for processing. Figure S1 shows the study flow chart.

The first pus swab was used to make gram stain smears while the second one was inoculated onto Blood agar and Mannitol-salt agar and incubated at 35–37°C for 24 hours for recovery of staphylococci. Isolates were initially identified using standard microbiological procedures based on colony morphology on Blood agar which are creamy to golden yellow in color with or without hemolysis, and on Mannitol-salt agar by formation of yellow zone around colonies. For phenotypic confirmation of *S. aureus*, the catalase test was done followed by slide and tube coagulase tests, as well as DNAse test.

### Drug Susceptibility Testing (DST)

A standard disc diffusion technique for antimicrobial susceptibility testing was performed as recommended by the Clinical and Laboratory Standard Institute (CLSI) [28] on Mueller-Hinton agar (Biolab®, Hungary). Standard antimicrobial disks (Biolab®, Hungary) set were ampicillin (10 µg), oxacillin (1 µg), trimethoprim-sulphamethoxazole (1.25/23.75 µg), tetracycline (30 µg), ciprofloxacin (5 µg), chloramphenicol (30 µg), gentamicin (10 µg), erythromycin (15 µg), clindamycin (2 µg), and vancomycin (30 µg). The plates were incubated at 37°C for 24 hours. All *S. aureus* isolates with zone of inhibition on cefoxitin (30 µg) disk of ≤21 mm were phenotypically considered MRSA. For determining inducible clindamycin resistance, clindamycin disk (2 µg) and erythromycin disk (15 µg) were placed side by side approximately 15–26 mm apart. Flattening of the zone of inhibition adjacent to the erythromycin disk was regarded as a positive D-test (MLSb phenotype i.e. inducible resistance of clindamycin to other macrolides namely, lincosamine and streptogramin B) [28].

Apart from conventional methods, isolates confirmation and drug susceptibility testing were done using the Phoenix Automated instrument (Becton-Dickson, Sparks Maryland) as per manufacturer’s instruction.

### Molecular Assays

All PCR primers used in this study are described in Table S1.

#### i) Detection of the *nuc* gene

*S. aureus* chromosomal DNA was extracted as previously described [21] and species confirmed through PCR-amplification and identification of a 270 bp amplicon of the *S. aureus* thermonuclease (*nuc*) gene [29], [30]. The PCR program comprised of initial denaturation at 94°C for 5 minutes followed by 30 cycles of denaturation at 94°C for 45 seconds, primer annealing at 50°C for 45 seconds, extension at 72°C for 45 seconds, and a final extension at 72°C for 10 minutes. The amplicons were analyzed by electrophoresis on a 2% agarose gel (Table S1). Images were captured and retrieved using the Bio-imager (UVP, Upland, California, USA).

#### ii) Detection of the mecA and pvl genes

Confirmation of MRSA was based on PCR-detection of the *mecA* gene (usually the gold standard) through amplification and identification of a 162 bp amplicon upon agarose gel electrophoresis. Presence of the *pvl* gene was also determined by PCR-amplification and detection of a 433 bp region overlapping the *lukS-PV* and *lukF-PV* genes. Both *mecA* and *pvl* genes were detected through a multiplex PCR as previously described [31]. The PCR program comprised of initial denaturation at 94°C for 5 minutes followed by 30 cycles of denaturation at 94°C for 30 seconds, primer annealing at 53°C for 30 seconds, extension at 72°C for 45 seconds, and a final extension at 72°C for 10 minutes. The amplicons were analyzed by electrophoresis on a 2% agarose gel (Table S1).

#### iii) SCC*mec* genotyping

The multiplex PCR we followed in typing SCC*mec* types I–V was described previously [22]. However, for improved efficiency, in this study we introduced minor modifications in the protocol such as using two primer pairs in each case (i.e. ccrC and 5R; αβ and IS1272). The PCR program comprised of initial denaturation at 94°C for 5 minutes followed by 30 cycles of denaturation at 94°C for 45 seconds, primer annealing at 50°C for 45 seconds, extension at 72°C for 45 seconds, and a final extension at 72°C for 10 minutes. The amplicons were analyzed by electrophoresis on a 2% agarose gel (Table S1). Following PCR, interpretation of amplicons was based on number and size of band(s) for the four target amplicons upon agarose gel electrophoresis; where no band was observed the respective isolate was regarded as “non-typeable”. Repeated testing and optimization of PCR conditions were done in which the results of non-typeable isolates results were reproducible (i.e. no amplification following repeated PCRs).

#### iv) *spa* genotyping and sequence analysis

To determine the genetic lineages of *S. aureus,* the x- region of the *spa* gene (200 bp to 400 bp) was amplified by PCR using the method established before [25]. The PCR program comprised of initial denaturation at 94°C for 5 minutes followed by 30 cycles of denaturation at 94°C for 45 seconds, primer annealing at 50°C for 45 seconds, extension at 72°C for 45 seconds, and a final extension at 72°C for 10 minutes. The amplicons were analyzed by electrophoresis on a 2% agarose gel (Table S1). The PCR products were purified with the QIAquick PCR purification kit (Qiagen, Hilden, Germany) as per the manufacturer’s instructions, and both strands were sequenced by ACGT (Wheeling, IL, USA) using the same forward and reverse primers (Table S1). To determine *spa* types, sequences were submitted to the spaTyper data base (http://fortinbras.us/cgi-bin/spaTyper/spaTyper.pl) and *spa* lineages matching to the query sequence determined. The data was also matched with the Ridom SpaServer (Ridom GmbH, Wurzburg, Germany) (http://spa.ridom.de/). Chromatograms for unique sequences (i.e. lacking hits) were submitted to the Ridom SpaServer (http://spa.ridom.de/) to assess novelty of lineages.

### Quality Control

Standard aseptic sample collection and processing measures were strictly adhered. For phenotypic identification of *S. aureus*; *S. aureus* ATCC 25923 and *S. epidermidis* ATCC 12228 were used as positive and negative controls, respectively. For genotyping, *S. aureus* ATCC 29213 (mecA-negative, PVL-negative), and ATCC 43300 (mecA-positive, PVL-negative) were used.

### Data Analysis

Variables from demographic, clinical and laboratory data were entered into excel spreadsheet, cleaned and exported to STATA software version 11 (College Station, Texas, USA) for analysis according to the study objectives. Continuous variables were summarized as mean (± standard deviation). Categorical variables were described as proportion and were analyzed to compare the significance of difference in distribution by using Chi-square test or Fischer’s exact test where appropriate. All variables with association using univariate logistic regression were subjected to multivariate logistic regression analysis. Odds ratio with respective 95% confidence interval was used to measure the strength of association between the surgical site infection and predictive variables. Variables with *p-*value less than 0.05 were considered as independent predictors of surgical site infections.

### Study Clearance and Ethical Considerations

This study got ethical clearance from the Institutional Review Board (IRB) of Makerere University College of Health Sciences (REC REF # 2011–183), Mulago Hospital Research Committee (MREC # 125) and Uganda National Council for Science and Technology (UNCST) (REF # HS 1080). A written informed consent (or assent) was obtained from each patient/caretaker. All patient information was kept confidential and anonymous using codes. Results for antimicrobial susceptibility testing were promptly reported to the attending physician for patient care.

## Results

Three hundred and fourteen (314) patients with clinical SSI were enrolled in this study of which 76.1% (239/314) were female with median age of 25±13.14 years. *S. aureus* was isolated from 64 patients (20.4%, 64/314, one isolate per patient) of which 24 isolates (37.5%, 24/64) were MRSA (Figures S2 and S3).

### Antimicrobial Resistance Patterns among MSSA and MRSA

MRSA isolates were predominantly multi-drug resistant (i.e. resistant to three or more antimicrobial classes) compared to MSSA, with significant difference in oxacillin [91.7% (22/24) and 5.0% (2/40)], gentamicin [33.3% (8/24) and 10.0% (4/40)], ciprofloxacin [50.0% (12/24) and 17.5% (7/40)] and chloramphenicol [37.5% (9/24) and 2.5% (1/24)] among MRSA and MSSA isolates respectively (Table 1). All *S. aureus* isolates were susceptible to vancomycin. Two MRSA isolates were misidentified as MSSA by the disc diffusion method using oxacillin (1 µg) and cefoxitine (30 µg) discs, but were confirmed to be MRSA upon *mecA-*PCR. Their respective zones of inhibition were 22 mm and 23 mm for cefoxitin, and 13 mm for oxacillin for both.

**Table 1:** Comparison of antimicrobial resistance patterns between MSSA and MRSA.

Antimicrobial	MSSA = 40n (%)	MRSA = 24n (%)	chi2	P-value
Ampicillin	40 (100.0)	24 (100.0)	–	–
Oxacillin	2 (5.0)	22 (91.7)	48.173	0.000
Cotrimoxazole	34 (85.0)	23 (95.8)	1.807	0.179
Gentamicin	4 (10.0)	8 (33.3)	6.431	0.040
Tetracycline	15 (37.5)	12 (50.0)	2.432	0.488
Ciprofloxacin	7 (17.5)	12 (50.0)	7.721	0.021
Chloramphenicol	1 (2.5)	9 (37.5)	14.622	0.001
Erythromycin	14 (35.0)	16 (66.7)	6.285	0.099
Clindamycin	14 (35.0)	12 (50.0)	1.482	0.477
Vancomycin	0 (0.0)	0 (0.0)	–	–

### Genetic Lineages of *S. aureus* based on *spa* Typing and their Distribution in Different Wards

A total of 14 *spa* lineages were determined. One third of these fell in two major lineages namely t645 and t4353, which accounted for 17.2% (11/64) and 15.6% (10/64), respectively. The major lineages were found to be clonally circulating in all the surgical wards whereas lineages t064, t355, and t4609 were confined to obstetrics and gynecology wards. A new *spa* type namely t10277 was also identified in this study from MSSA isolate (Table 2). The association of these *spa* types with MSSA and MRSA are shown in Table S2. Apparently, 11 isolates had sequent repeats with no identity in the *spa* server (i.e. novel clones) and the *spa* gene was not detected in four isolates on sequencing, despite a repeated reactions.

**Table 2:** Genetic lineages of S. aureus based on spa typing and their distribution in different wards.

spa types	Sequent repeats	Surgical Wards			
		Obstetrics & Gynecology	General surgery	Orthopedic	Total
t645	E1:G1:M1:J1:H2:M1 13∶12:17∶23:18∶17	7 (63.6%)	3 (27.3%)	1 (9.1%)	11 (100.0%)
t4353	U1:J1:G1:F1:F1:G1:M1:D1:M1:G1:M1 07∶23:12∶21:21∶12:17∶20:17∶12:17	4 (40.0%)	4 (40.0%)	2 (20.0%)	10 (100.0%)
t064	Y1:H1:G1:C1:M1:B1:Q1:B1:L1:O111∶19:12∶05:17∶34:24∶34:22∶25	4 (100.0%)	0 (0.0%)	0 (0.0%)	4 (100.0%)
t084	U1:J1:G1:B1:B1:G1:G1:J1:A1:G1:J107∶23:12∶34:34∶12:12∶23:02∶12:23	1 (25.0%)	3 (75.0%)	0 (0.0%)	4 (100.0%)
t355	U1:J2:G1:M1:K1:K1:P1:N1:S1:G107∶56:12∶17:16∶16:33∶31:57∶12	4 (100.0%)	0 (0.0%)	0 (0.0%)	4 (100.0%)
t3772	Z1:G1:U2:D1:M1:G1:V2:V2:U204∶12:41∶20:17∶12:48∶48:41	2 (66.7%)	1 (33.3%)	0 (0.0%)	3 (100.0%)
t4609	Y1:H1:G1:C1:M1:B1:Q1:B1:M1:B1:L1:O111∶19:12∶05:17∶34:24∶34:17∶34:22∶25	3 (100.0%)	0 (0.0%)	0 (0.0%)	3 (100%)
t037	W1:G1:K1:A1:O1:M1:Q115∶12:16∶02:25∶17:24	1 (50.0%)	1 (50.0%)	0 (0.0%)	2 (100.0%)
t189	U1:J1:G1:F1:M1:B1 07∶23:12∶21:17∶34	1 (50.0%)	1 (50.0%)	0 (0.0%)	2 (100.0%)
t127	U1:J1:F1:K1:B1:P1:E1 07∶23:21∶16:34∶33:13	1 (100.0%)	0 (0.0%)	0 (0.0%)	1 (100.0%)
t130	A2:B1:E1:M1:B1:K1:B109∶34:13∶17:34∶16:34	1 (100.0%)	0 (0.0%)	0 (0.0%)	1 (100.0%)
t1456	X1:K1:A1:O108∶16:02∶25	1 (100.0%)	0 (0.0%)	0 (0.0%)	1 (100.0%)
t2029	W1:G1:A1:O1:M1:Q115∶12:02∶25:17∶24	0 (0.0%)	1 (100.0%)	0 (0.0%)	1 (100.0%)
t10277	X1:K1:A1:K1:B1:B1:M1:B1:E108∶16:02∶16:34∶34:17∶34:13	0 (0.0%)	1 (100.0%)	0 (0.0%)	1 (100.0%)
Unique*	Different in each isolate	7 (63.6%)	4 (36.4%)	0 (0.0%)	11 (100.0%)
ND+	–	2 (50.0%)	1 (25.0%)	1 (25.0%)	4 (100.0%)
Total	–	40 (62.5%)	20 (31.3%)	4 (6.3%)	64 (100.0%)

Pearson Chi-Square 32.65, p-value 0.008.

* Sequence repeats with no identity in the spa server.

+ Not detected.

### SCC*mec* Types among MRSA from SSI

The most predominant MRSA genotypes were SCC*mec* type V (33.3%, 8/24), followed by type I (16.7%, 4/24); SCC*mec* types II and IV accounted for 8.3% (2/24) and 4.2% (1/24), respectively. Apparently, 25% of the isolates were non-typeable (NT) (Figure S4).

### Association between MRSA/MSSA with Clinical and Demographic Characteristics among Patients with SSI

There was a strong association between MRSA-SSI with infection extending to organ, cancer, and inducible clindamycin resistance (p-values = 0.005, 0.022 and 0.002, respectively) than MSSA-SSI (Table 3). Inducible clindamycin resistance accounted for 17.2% (11/64) of all *S. aureus*; this was more among MRSA compared to MSSA isolates [72.7% (8/11) and 27.3% (3/11), respectively, p = 0.002)] (Table 3). On multivariate logistic regression analysis, cancer and inducible clindamycin resistance remained as independent predictors of MRSA-SSI (Table 4).

**Table 3:** Association between MRSA/MSSA with clinico-demographic characteristics among patients with SSI.

Variable	Patients with *S. aureus* SSI		OR [95% CI]	p-value
	MSSA (n = 40	MRSA (n = 24)		
**Age**				
≤30 years	27 (61.3)	17 (38.6)	1	
>30 years	13 (65.0)	7 (35.0)	0.9 [0.2–2.9]	0.7806
**Sex**				
Male	8 (72.70	3 (27.3)	1	
Female	32 (60.4)	21 (39.6)	1.75 [0.4–11.3]	0.4413
**Wards**				
Obstetrics & Gynecology	24 (60.0)	16 (40.0)	1	
General surgery	13 (65.0)	7 (35.0)	0.8 [0.2–2.8]	0.7073
Orthopedic	3 (75.0)	1 (25.0)	0.5 [0.01–7.0]	0.5569
**Operation**				
Elective	14 (70.0)	6 (30.0)	1	
Emergency	26 (40.1)	18 (59.1)	1.6 [0.5–6.1]	0.4034
**Extent of infection**				
Deep	24 (77.4)	7 (22.6)	1	
Organ	2 (25.0)	6 (75.0)	10.3 [1.3–117.2]	0.0050
Superficial	14 (56.0)	11 (44.0)	2.7 [0.7–10.1]	0.0880
**Cancer**				
No	40 (65.6)	21 (34.4)		
Yes	0 (0.0)	3 (100.0)	–	0.0220
**HIV serostatus**				
Negative	13 (72.2)	5 (27.8)	1	
Positive	10 (66.7)	5 (33.3)	1.3 [0.2–7.4]	0.7295
**Colonization with ** ***S. aureus***				
No	37 (63.8)	21 (36.2)	1	
Yes	3 (50.0)	3 (50.0)	1.8 [0.2–14.2]	0.5065
**Length of hospital stay (admission to discharge)**				
≤7 days	19 (76.0)	6 (24.0)	1	
>7 days	21 (53.9)	18 (46.1)	2.7 [0.8–10.0]	0.0741
**Presence of ** ***pvl***				
No	23 (57.5%)	17 (42.5%)	1	
Yes	17 (70.8%)	7 (29.2%)	0.56 [0.16–1.8]	0.286
**Inducible clindamycin resistant (D test +)**				
No	23 (79.3%)	6 (20.7%)	1	
D-test negative	14 (58.3%)	10 (41.7%)	2.74 [0.70–11.18]	0.098
D-test positive	3 (27.3%)	8 (72.7%)	10.22 [1.66–73.52]	0.002

**Table 4:** Multivariate logistic regression analysis of factors associated with MRSA SSI.

Predictor variable	OR	95% CI	p-value
Extent of SSI (organ involvement)	0.96	0.39–2.35	0.921
Duration from admission to discharge, median days	1.01	0.96–1.08	0.599
Inducible clindamycin resistant	0.34	0.16–0.74	0.007

Additionally, of the 64 patients with S. aureus SSI, six were colonized by S. aureus in the nose and only one was concurrently infected and colonized with the same MRSA strain (SCCmec V and spa type t657). Thus, the overall nasal colonization rate with S. aureus was 9.4% (6/64) while nasal colonization rate with the same strain causing SSI was 1.6% (1/64).

Finally, the *pvl* gene was found in 37.5% of *S. aureus* (42.5% of MSSA and 29.2% of MRSA) (Table 3).

## Discussion

The prevalence of *S. aureus* in patients with SSI in this study (20.4%) was lower than that found in other studies (28.7% and 28.6%) [9], [11]. However, the proportion of MRSA among *S. aureus* was higher (37.5%) than previously reported from the same setting (31.5%) [11] and 4.8%–29.6%, 25%, and 18.8% from other studies in eight African countries, Jinja-Uganda and Mwanza-Tanzania, respectively [6], [9], [32]. The variability in prevalence may be influenced by infection control practices in different settings.

The finding that MRSA is associated with multi drug resistance compared to MSSA in this study have also been reported in other studies [6], [33], [34]. Similar to another study in the same region [35], *S. aureus* exhibiting phenomenal inducible clindamycin resistance (D-test positive) accounted for 17.2%, more were found in MRSA compared to MSSA isolates (p = 0.002)**.** This implies that both clindamycin and erythromycin cannot be used in these patients.

In this study, the proportion of females with SSI was higher compared to other studies [9], [14], [32], whereby females accounted for 40% to 60.0%. This was because more than half of patients were from obstetrics and gynecology wards. Similar to other studies [9], [14], [32], the median age was variably ranging from 30 to 60 years.

On univariate analysis, there was a statistical significant association between MRSA-SSI with infections involving organ, cancer, and inducible clindamycin resistance (p = 0.005, 0.022, and 0.002, respectively) in comparison with MSSA-SSI. Furthermore, on multivariate logistic regression analysis, cancer and inducible clindamycin resistance remained as independent predictors of MRSA-SSI. Other studies have reported that long duration of hospital stay and presence of co-morbid conditions have been found to be more associated with MRSA-SSI [1], [9], [14].

Of the 14 *spa* lineages found in this study, one third fell among two major lineages (t645 and t4353) and were found to be clonally circulating in all the surgical wards; on the other hand, t064, t355, and t4609 were confined to obstetrics and gynecology wards. This calls for strengthening of infection control practices at Mulago National Hospital to prevent this clonal spread of *S. aureus* lineages.

There seems to be diversity between *spa* types in different regions and different countries. Indeed none of the *spa* lineages in the index study were found in two studies in the USA and UK [16], [36] whereas lineages t037, t064, t084, t127, t355, and t3772 found in this study were similar to those previously reported from Nigeria [34]. t064, also found in this study was reported in Mwanza, Tanzania [15]. The variability in *spa* lineages across regions emphasizes the need for region wide infection control surveillance.

In this study, SCC*mec* type V was the most predominant among MRSA, followed by SCC*mec* types I, IV and III. The predominance of SCC*mec* type V was previously reported among isolates from the burns units at Mulago National Hospital [37] and in another study in Nigeria [34]. However, in these other studies SCC*mec* types I and III were also predominant. SCC*mec* types I, II, and III are usually ascribed to HA-MRSA isolates whereas SCC*mec* types IV and V are ascribed to CA-MRSA (which accounted for 29.1% and 45.8%, respectively, of the MRSA isolates in the current study) [23]. Indeed, the findings from Uganda and Nigeria may suggest presence of mixed CA-MRSA and HA-MRSA genotypes in hospital settings, supporting the notion that there is a changing epidemiology reflected by community associated SCC*mec* genotypes being now more associated with hospital infections [38]. Nevertheless, based on the protocol by Boye *et al* (2007) [22], a quarter of the isolates in this study were non-typeable despite optimization of laboratory methods suggesting they could belong to SCC*mec* types other than I–V (i.e. SCC*mec* types VI–VIII).

Of the 64 patients with *S. aureus* SSI, six were colonized by *S. aureus* in the nose and only one was concurrently infected and colonized with the same MRSA strain (SCC*mec* V and *spa* type t657). This suggests an external source of infection for the MRSA-SSI, either from healthcare workers, environment or other sources.

### Conclusions

SCC*mec* types V and I are the most prevalent MRSA genotypes. *S. aureus spa* lineages t645 and t4353 which were predominant are clonally circulating in all surgical wards, calling for strengthening of infection control practices at Mulago National Hospital. Studies to determine the sources of these lineages would be of interest, as well as studies comparing these isolates with other continental lineages.

## Supporting Information

Figure S1 **Study flow chart showing patient recruitment and laboratory procedures.**
(DOC)

Figure S2 **PCR-detection of the** ***nuc*** **gene confirming isolates as** ***S. aureus*** **.** Numbers 1 to 31 refer to patients’ isolates; +ve, positive control; -ve, negative control.
(DOC)

Figure S3 **Multiplex PCR in which the** ***mecA*** **and** ***pvl*** **genes were detected.** Numbers 1 to 16 refer to patient isolates; +ve, positive control; -ve negative control.
(DOC)

Figure S4 **Multiplex-PCR for SCC** ***mec*** **Typing.** Numbers 1 to 16 refer to patient isolates; –ve, negative control.
(DOC)

Table S1 **PCR primers used in this study.**
(DOC)

Table S2 ***S. aureus*** **clonal lineages based on** ***spa*** **typing among patients surgical site infection.** ^*^Sequence repeats with no identity in the spa server. ^+^Not detected.
(DOC)
