# Supplementary figures and images for: Molecular Characterization of Staphylococcus aureus from Patients with Surgical Site Infections at Mulago Hospital in Kampala, Uganda
Source: PLoS One. 2013 Jun 20;8(6):e66153. doi: 10.1371/journal.pone.0066153 (PMC3688721; doi:10.1371/journal.pone.0066153)

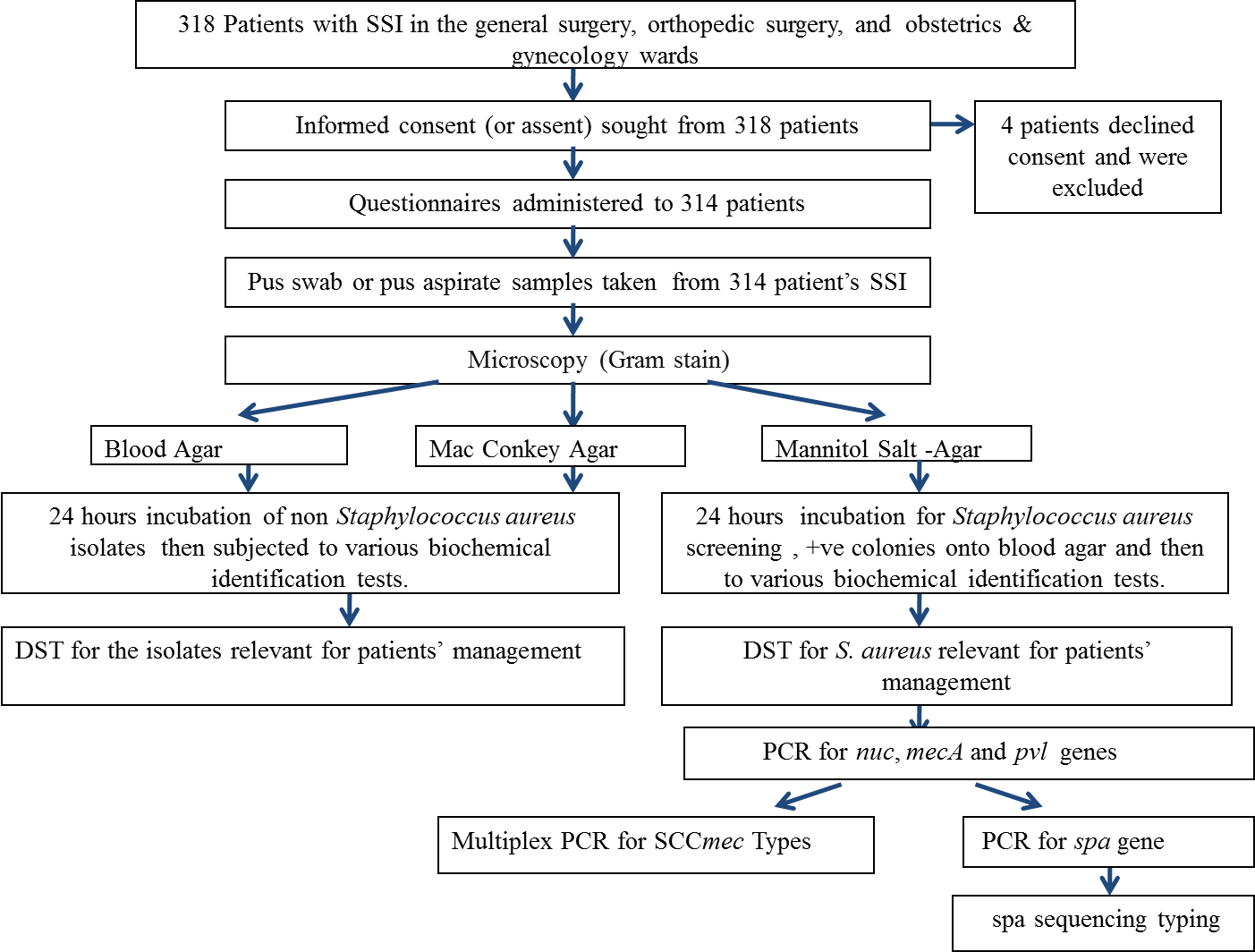

Supplement: Figure S1 — Study flow chart showing patient recruitment and laboratory procedures. (DOC) [file pone.0066153.s001.doc]

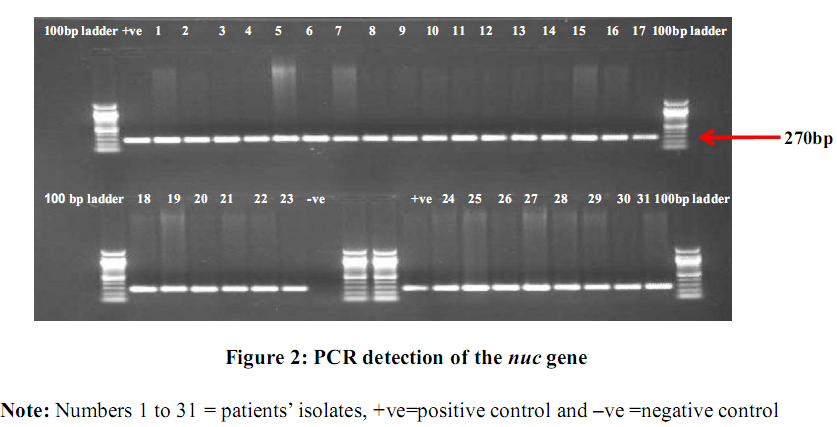

Supplement: Figure S2 — PCR-detection of the nuc gene confirming isolates as S. aureus . Numbers 1 to 31 refer to patients’ isolates; +ve, positive control; -ve, negative control. (DOC) [file pone.0066153.s002.doc]

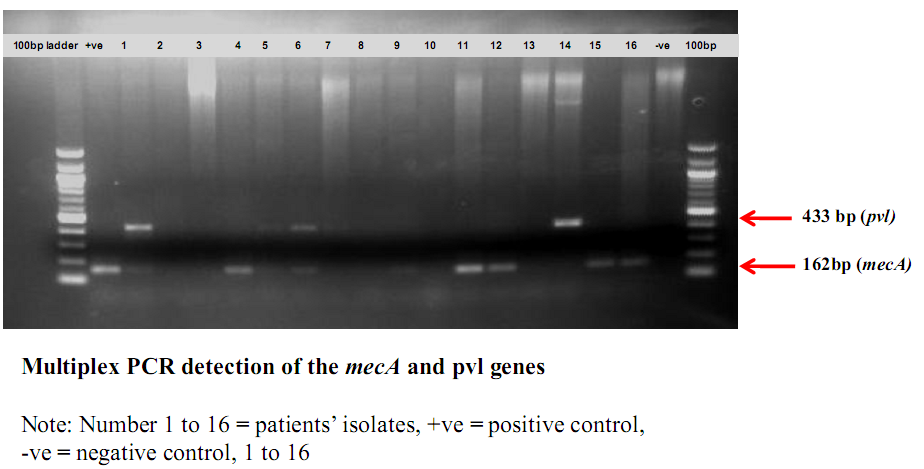

Supplement: Figure S3 — Multiplex PCR in which the mecA and pvl genes were detected. Numbers 1 to 16 refer to patient isolates; +ve, positive control; -ve negative control. (DOC) [file pone.0066153.s003.doc]

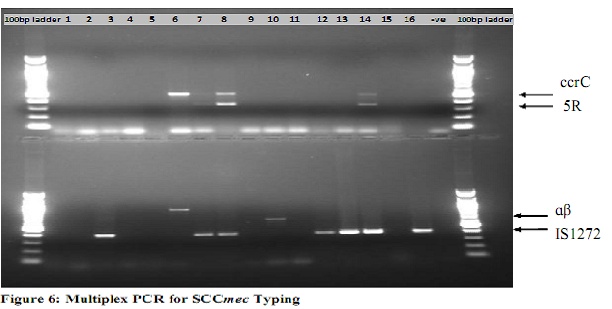

Supplement: Figure S4 — Multiplex-PCR for SCC mec Typing. Numbers 1 to 16 refer to patient isolates; –ve, negative control. (DOC) [file pone.0066153.s004.doc]
