# Supplementary material for: Molecular Characterization of Staphylococcus aureus from Patients with Surgical Site Infections at Mulago Hospital in Kampala, Uganda
Source: PLoS One. 2013 Jun 20;8(6):e66153. doi: 10.1371/journal.pone.0066153 (PMC3688721; doi:10.1371/journal.pone.0066153)
Supplement: Table S1 — PCR primers used in this study. (DOC) [file pone.0066153.s005.doc]

| **Target** | **Primer name** | **Synthesizing company** | **Primer sequence (5′-3′)** | **Product size (bp)** | **Reference(s)** |
| --- | --- | --- | --- | --- | --- |
| *nuc* | Nuc1 | Eurofins-MWGOperon, Ebersberg, Germany | 5'-GCGATTGATGGTGATA CGGTT-3' | 270 | 29, 30 |
|  | Nuc2 |  | 5'-AGCCAAGCCTTGACGAACTA AAGC-3' |  |  |
| *mecA* | P4 | Integrated DNA Technologies, Leuven, Belgium | 5' -TCCAATTACAACTTCACCAGG’-3 | 162 | 31 |
|  | P7 |  | 5’-CCACTTCATATCTTGTAACG-3‘ |  |  |
| PVL | *luk-PV-1* | Invitrogen, Stockholm, Sweden | 5’-ATCATTAGGTAAAATGTCTGGACATGATCCA-3’ | 433 | 31 |
|  | *luk-PV-2* |  | 5’-GCATCAAGTGTATTGGATAGCAAAA GC-3’ |  |  |
| spa | spaF | Integrated DNA Technologies, Leuven, Belgium | 5’- AGA CGA TCC TTC GGT GAG- 3’ | 200-400 | 25 |
|  | spaR |  | 5’- GCTTTTGCAATGTCATTTACTG-3’ |  |  |
| *ccrC* | *ccrCF* | Integrated DNA Technologies, Leuven, Belgium | 5’-CGTCTATTACAAGATGTTAAGGATAAT-3’ | 518 | 22 |
|  | *ccrCR* |  | 5-CCTTTATAGACTGGATTATTCAAAATAT-3’ |  |  |
| *ɑβ* | Beta | Integrated DNA Technologies, Leuven, Belgium | 5’-ATTGCCTTGATAATAGCCYTCT-3’ | 937 |  |
|  | Alpha3 |  | 5’-TAAAGGCATCAATGCACAAACACT-3’ |  |  |
| *IS1272* | 1272F1 | Integrated DNA Technologies, Leuven, Belgium | 5’-GCCACTCATAACATATGGAA-3’ | 415 |  |
|  | 1272R1 |  | 5’-CATCCGAGTGAAACCCAAA-3’ |  |  |
| mecA–IS431 | 5RmecA | Integrated DNA Technologies, Leuven, Belgium | 5’-TATACCAAACCCGACAACTAC-3’ | 359 |  |
|  | 5R431 |  | 5’-CGGCTACAGTGATAACATCC-3’ |  |  |
