# Supplementary material for: Molecular Characterization of Staphylococcus aureus from Patients with Surgical Site Infections at Mulago Hospital in Kampala, Uganda
Source: PLoS One. 2013 Jun 20;8(6):e66153. doi: 10.1371/journal.pone.0066153 (PMC3688721; doi:10.1371/journal.pone.0066153)
Supplement: Table S2 — S. aureus clonal lineages based on spa typing among patients surgical site infection. *Sequence repeats with no identity in the spa server. +Not detected. (DOC) [file pone.0066153.s006.doc]

| ***spa* type** | **MSSA** | | **MRSA** | | **Total** | |
| --- | --- | --- | --- | --- | --- | --- |
|  | Frequency | % | Frequency | % | Frequency | % |
| t645 | 9 | 81.8 | 2 | 18.2 | 11 | 100.0 |
| t4353 | 9 | 90.0 | 1 | 10.0 | 10 | 100.0 |
| t064 | 3 | 75.0 | 1 | 25.0 | 4 | 100.0 |
| t084 | 4 | 100.0 | 0 | 0.0 | 4 | 100.0 |
| t355 | 4 | 100.0 | 0 | 0.0 | 4 | 100.0 |
| t3772 | 1 | 33.3 | 2 | 66.7 | 3 | 100.0 |
| t4609 | 1 | 33.3 | 2 | 66.7 | 3 | 100.0 |
| t037 | 0 | 0.0 | 2 | 100.0 | 2 | 100.0 |
| t189 | 2 | 100.0 | 0 | 0.0 | 2 | 100.0 |
| t127 | 0 | 0.0 | 1 | 100.0 | 1 | 100.0 |
| t130 | 1 | 100.0 | 0 | 0.0 | 1 | 100.0 |
| t1456 | 0 | 0.0 | 1 | 100.0 | 1 | 100.0 |
| t2029 | 0 | 0.0 | 1 | 100.0 | 1 | 100.0 |
| t10277 | 1 | 100.0 | 0 | 0.0 | 1 | 100.0 |
| Unique^*^ | 5 | 45.5 | 6 | 54.5 | 11 | 100.0 |
| ND^+^ | 0 | 0.0 | 4 | 100.0 | 4 | 100.0 |
| Total | 40 | 62.5 | 24 | 37.5 | 64 | 100.0 |
